# Noncanonical placental Fc receptors: What is their role in modulating transplacental transfer of maternal IgG?

**DOI:** 10.1371/journal.ppat.1007161

**Published:** 2018-08-30

**Authors:** David R. Martinez, Genevieve G. Fouda, Xinxia Peng, Margaret E. Ackerman, Sallie R. Permar

**Affiliations:** 1 Department of Molecular Genetics and Microbiology, Duke University Medical Center, Durham, North Carolina, United States of America; 2 Duke Human Vaccine Institute, Duke University Medical Center, Durham, North Carolina, United States of America; 3 Department of Pediatrics, Duke University Medical Center, Durham, North Carolina, United States of America; 4 Bioinformatics Research Center, North Carolina State University, Raleigh, North Carolina, United States of America; 5 Thayer School of Engineering, Dartmouth College, Hanover, New Hampshire, United States of America; McGill University, CANADA

## Introduction

The transplacental transfer of maternal Immunoglobulin G (IgG) to the fetus is critical for protection against infectious diseases in the first year of life [1]. Maternal protective IgG is transferred from the maternal to the fetal circulatory system via the placenta, and this process begins in the first trimester of pregnancy [2]. By 37–40 weeks of gestation, maternal passively acquired IgG concentrations in newborns can exceed maternal IgG serum levels in normal pregnancies [3–7]. Yet, the molecular mechanisms of transplacental transfer of maternal IgG remain poorly understood. In order to reach the fetal circulatory system, maternal IgG must traverse three distinct placental anatomical barriers: (1) the syncytiotrophoblast cell barrier, (2) the villous stroma containing placental fibroblasts and Hofbauer cells, and (3) fetal endothelial cells. It is well established that IgG crosses the syncytiotrophoblast by binding to the canonical IgG shuttle receptor: Fragment crystallizable (Fc) receptor neonatal (FcRn) [2, 8]. However, how maternal IgG traverses the subsequent placental barriers is not completely understood, as they do not express FcRn, yet recent RNAseq analyses have shown that Fcγ receptors, including FcγRIIIa, FcγRIIa, FcγRIIb, and FcγRI, are expressed in term placentas [9]. However, it should be cautioned that it is not yet known if these noncanonical placental FcRs play a role, if at all, in the transplacental transfer of maternal IgG.

A deeper understanding of the molecular mechanism(s) of IgG binding to placentally expressed Fc receptors could be important (1) for the design of novel maternal IgG-based therapeutics and vaccines with optimal transplacental transfer efficiency, with the ultimate goal of increasing infant protection against congenital and neonatal infectious diseases, and (2) to optimize the Fc region of immunomodulatory IgG monoclonal antibody therapeutics for blunted transplacental transfer to potentially reduce the transplacental transport of maternal self-reactive IgG in women with autoimmune disorders.

## Transplacental transfer activity of FcRn and its molecular interactions with IgG

Human FcRn consists of alpha and beta subunits that assemble to form a membrane-bound heterodimer receptor [8, 10]. FcRn is primarily expressed in intracellular endosomes in placental syncytiotrophoblast cells, and it shuttles maternal IgG from the apical side to the basolateral membrane [10]. In the proposed model of the transplacental transfer of IgG in syncytiotrophoblast cells, IgG is first phagocytosed into endosomes containing membrane-bound FcRn [10]. Upon exposure to endosome acidification from pH 7.4 to pH 6, IgG Fc binds to FcRn via electrostatic interactions [2, 10]. Next, the endosome is released on the basolateral side of the syncytiotrophoblast, and once the FcRn:IgG complex is extracellularly exposed to pH 7.4, the complex dissociates, releasing IgG into the villous stroma [10].

The acidic pH-dependent interaction of IgG and placental FcRn is modulated by the formation of salt bridges between basic amino acid residues H310 (IgG1 subclass amino acid numbering convention) in the constant heavy 2 (CH2) domain and H435 and H436 in the CH3 domain of the Fc region, and they interact with acidic amino acid residues E117, E132, and D137 in the beta subunit of FcRn [11]. While crystallography data demonstrate that amino acid residues within the CH2 and CH3 domains of IgG Fc interact with outer amino acid residues in the beta subunit of FcRn, mutational analyses suggest that additional amino acid residues outside the binding interface of IgG and Fc are also important for binding affinity [12]. For example, single amino acid residue substitutions of T307, E380, and N434 to alanine residues result in up to a 3-fold increase in binding to FcRn and up to a 12-fold increase when alanines at these positions are introduced in combination [12]. Thus, amino acid residues outside the binding interface of IgG Fc and FcRn may also be important for binding. Furthermore, recent studies demonstrated that IgG1 Fc region M428L and N434S mutations significantly improve the serum half-life of therapeutic IgG in adults by increasing binding affinity to FcRn [13]. Yet, the potential impact of these Fc region mutations on transplacental IgG transfer efficiency remains unknown and should be investigated.

## The potential role of FcγRIII and FcγRII in transplacental IgG transfer

**Fig 1:**
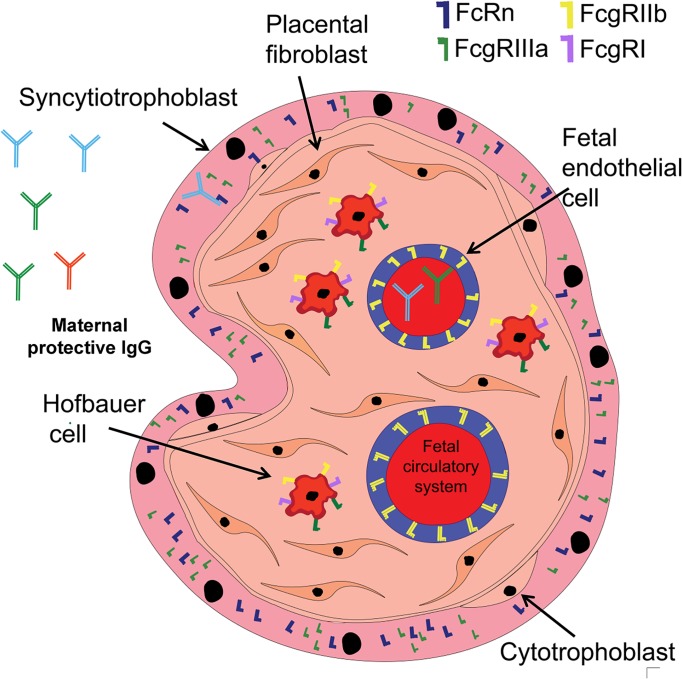
The distribution of Fc receptor expression in placental villous trees. Maternal IgG present in the intervillous space crosses through distinct cell barriers, including syncytiotrophoblasts, the villous stroma which contains Hofbauer cells and fibroblasts, and fetal endothelial cells. Placental FcRn and FcγRIIIa are expressed in the outermost cell barrier of the villous tree: the syncytiotrophoblast. Hofbauer cells located in the villous stroma express FcγRI, FcγRIIb, and FcγRIIIa. Fetal endothelial cells express FcγRIIb but not FcRn. Fc, Fragment crystallizable; FcRn, Fc receptor neonatal; FcγRI, Fragment crystallizable gamma RI; FcγRIIb, Fragment crystallizable gamma RIIb; FcγRIIIa, Fragment crystallizable gamma RIIIa; IgG, Immunoglobulin.

The molecular mechanisms of the transplacental IgG transfer beyond the syncytiotrophoblastic cell barrier remain poorly understood. Importantly, placental cell barriers internal to the syncytiotrophoblast layer, including fibroblasts and Hofbauer cells of the villous stroma, and fetal endothelial cells, do not express the canonical placental IgG shuttle receptor FcRn (Fig 1). Yet, these downstream placental cell barriers express noncanonical Fc receptors. For example, Hofbauer cells express FcγRIII, FcγRII, and FcγRI but not FcRn, whereas placental fibroblasts are not known to express any Fcγ receptors. Finally, while the fetal endothelial cell—the final cell barrier that maternal IgG crosses before reaching the fetal circulatory system—does not express FcRn, it does express FcγRII [2, 14, 15]. Previous studies that examined the transplacental IgG transfer activity of FcγRIIb showed that endocytosed IgG colocalizes with FcγRIIb in endothelial cell endosomes [14–16]. Intriguingly, both IgG-bound FcγRIIb and free FcγRIIb were observed inside these endosomes, suggesting that this low-IgG-affinity receptor may play a role in the shuttling of maternal IgG into the lumen of fetal endothelial vessels [15]. In addition, FcγRIIIa and FcγRI, when engaged with IgG, can signal through Ig tyrosine-activating motif (ITAM), whereas FcγRIIb signals through Ig tyrosine-inhibition motif (ITIM) [17]. However, the Fc receptor IgG-dependent activation or inhibition of downstream placental cell signaling pathways as they relate to transplacental IgG transfer is unknown.

Placental FcγRIII expression, as defined by immunohistochemistry analyses, is largely localized to syncytiotrophoblast cells in the placental villous tree (Fig 1) [1,18–21]. FcγRIIIa tryptophan amino acid residues interact with invariant prolines and the interchain disulfide bridge of the CH2 domains of IgG, and these amino acid residues are conserved among all four IgG subclasses in humans [22]. Specific Fc region amino acid residues are similarly required for binding to FcγRIIb. In fact, mutational analyses suggest that several Fc region amino acid residues within the CH2 domain can alter binding to FcγRIIb [23]. In addition to Fc region outer-surface contact amino acid residues, the IgG Fc region N-linked glycosylation (N297 in IgG1) is important for binding affinity to FcγRs [24], and this Fc region glycan is conserved among the four IgG subclasses [22]. For example, an Fc region digalactosylated glycan increases the affinity for FcγRIIIa, whereas fucose decreases the binding affinity [25, 26]. Therefore, both Fc region amino acid residues and Fc region N-linked glycans mediate IgG binding to placental Fcγ receptors, raising the question of whether modulation of these IgG Fc characteristics could impact placental IgG transfer efficiency.

## Fc receptor polymorphisms and their potential role on transplacental IgG transfer activity

To date, no common single nucleotide polymorphisms (SNPs) have been identified for human FcRn [27]. However, allelic variation near the FcRn promoter has been implicated in altered transcriptional activity of FcRn in distinct human populations. As an example, variable number of tandem repeats (VNTR) variant 3 is more prevalent in Caucasian populations, and this allelic variation in the promoter is associated with increased transcriptional activity compared to VNTR variant 2 [27]. Nonsynonymous polymorphisms can also alter expression levels of FcγRIIb. For example, FcγRIIb can encode a nonsynonymous T > C SNP, which leads to either an I232 or T232 [28]. Interestingly, a T232 has been implicated in reduced localization to the membrane [29], which could potentially alter the ability of FcγRIIb to shuttle maternal IgG in fetal endothelial cells. Promoter SNPs have also been implicated in modulating gene expression levels of FcγRIIb [30], suggesting that SNPs regulate both the localization and expression levels of FcγRIIb. Similarly, nonsynonymous polymorphisms in FcγRIIIa, a nucleotide point mutation that leads to either F158 or V158, have been implicated in altering the binding affinity to IgG [31, 32]. For example, FcγRIIIa V158 has a stronger binding affinity for IgG subclasses compared to FcγRIIIa F158. Thus, polymorphisms among placentally expressed Fcγ receptors may play a role in transplacental IgG transfer.

## How could the placentally expressed Fc receptors be harnessed for improving infant health?

From our current understanding of the placental transfer of IgG, it remains unclear if placentally expressed Fc receptors [33], such as the Type II Fc receptor Dendritic Cell-Specific Intercellular adhesion molecule-3-Grabbing Non-integrin (DC-SIGN), or undiscovered IgG shuttle receptors, play a role, if at all, in transplacental IgG transfer. A deeper understanding of the molecular mechanisms of maternal IgG binding to alternative placental Fc receptors could be important for designing IgG-based therapeutics that increase infant protection against congenital viral infections and in early life. In fact, maternal passive immunization with polyclonal IgG during pregnancy has shown to be protective against congenital cytomegalovirus infection and is also being tested as a treatment strategy against congenital Zika syndrome [34, 35]. Therefore, future studies are needed to (1) define whether or not these noncanonical placentally expressed Fc receptors play a role in mediating the transplacental transfer of maternal IgG and (2) define how Fc receptor allelic variation impacts the transplacental transfer of maternal IgG. These data will guide the design of IgG-based maternal vaccines and therapeutics, fine-tuning transplacental transfer of IgG to improve maternal and infant health.
